# VR-prep improves visualization of medical data in augmented reality on smartphones

**DOI:** 10.1038/s41598-025-26856-7

**Published:** 2025-11-18

**Authors:** A. M. C. Böhner, A. M. Jacob, M. Bahaaeldin, L. Oelmeier, A. Isaak, C. C. Pieper, J. A. Luetkens, D. Kuetting

**Affiliations:** Clinic for Diagnostic and Interventional Radiology, University Hospital Bonn, Bonn, Germany

**Keywords:** Augmented reality, Medical imaging, Smartphones, CT, MRI, Image processing, Computed tomography, Three-dimensional imaging

## Abstract

Relaying complex anatomical information between clinicians and to patients is challenging in medical routine and might be improved via direct 3D visualization of image series in augmented reality (AR) in portable devices. We devised a workflow for displaying medical imaging data in augmented reality on smartphones, relying solely on open-source products: ‘Medical Imaging XR’ (MIXR) application, ‘3D Slicer’ software, and ‘Fiji’. We designed a pipeline for image transformation and data harmonization (VR-prep) to expedite and optimize the 3D rendering of DICOM series in MIXR. VR-prep significantly reduced the size of image files to be uploaded to MIXR, without sacrificing quality and readability of images. Due to reduced file size, upload and download times onto the mobile device were significantly lower. Our workflow is suitable for CT, MRI, US and PET-CT, whereas MIXR alone only permits CT data. VR-prep is user-friendly and allows for broader integration of medical imaging data sets in AR, to facilitate clinician to clinician and clinician to patient communication. VR-prep relies exclusively in open-source software making it viable to adopt in a broad range of applications from clinical decision-making, patient communication, and medical education.

**Supplementary Information:**

The online version contains supplementary material available at 10.1038/s41598-025-26856-7.

## Introduction

Augmented reality (AR) technology has garnered increasing attention in the field of medical imaging, offering promising opportunities for enhancing the visualization and interpretation of complex anatomical structures^1^. By optionally overlaying this imaging data onto the real-world environment, AR facilitates improved spatial understanding and communication among healthcare professionals, ultimately leading to more informed clinical decision-making^2,3^. In recent years, the proliferation of smartphones and tablets, equipped with powerful processors and high-resolution displays, holds the theoretical potential to give access to AR technology to a wider audience, but several hurdles remain^4,5^.

One of the key challenges in implementing AR for medical imaging lies in the seamless integration of imaging data into the AR environment. Traditional methods for visualizing medical images in AR often involve complex and time-consuming processes, requiring specialized software and expertise^6^. Furthermore, several of these applications are generated for head-mounted displays, which have associated to them relatively high costs. Despite recent advancements in software tools and mobile applications, there are still some difficulties in seamless integration of the wide variety medical imaging types for visualization in AR. There are however, commercial solutions that can address CT DICOM series and display them in AR on special head-mounted-displays or smartphones, e.g. via Medicalholodeck^TM^’s Medical Imaging XR (MIXR)^7–9^. MIXR is, at the time of writing, available free of charge for download for mobile devices.

In this paper, we present a time-efficient workflow called ‘VR-prep’ for visualizing medical imaging data in AR on smartphones and tablets, focusing only on the use of open-source products to ensure accessibility and affordability. VR-prep, which is a composition of codes designed for the software suite Fiji^10^, significantly decreases processing time and data size for the generation of 3D objects in MIXR, without compromising image quality. VR-prep also permits the inclusion to other imaging modalities than CT, including MRI, ultrasound, PET and even microscopy data.

## Results

### VR-prep within the workflow of extended reality data preparation

**Fig. 1:**
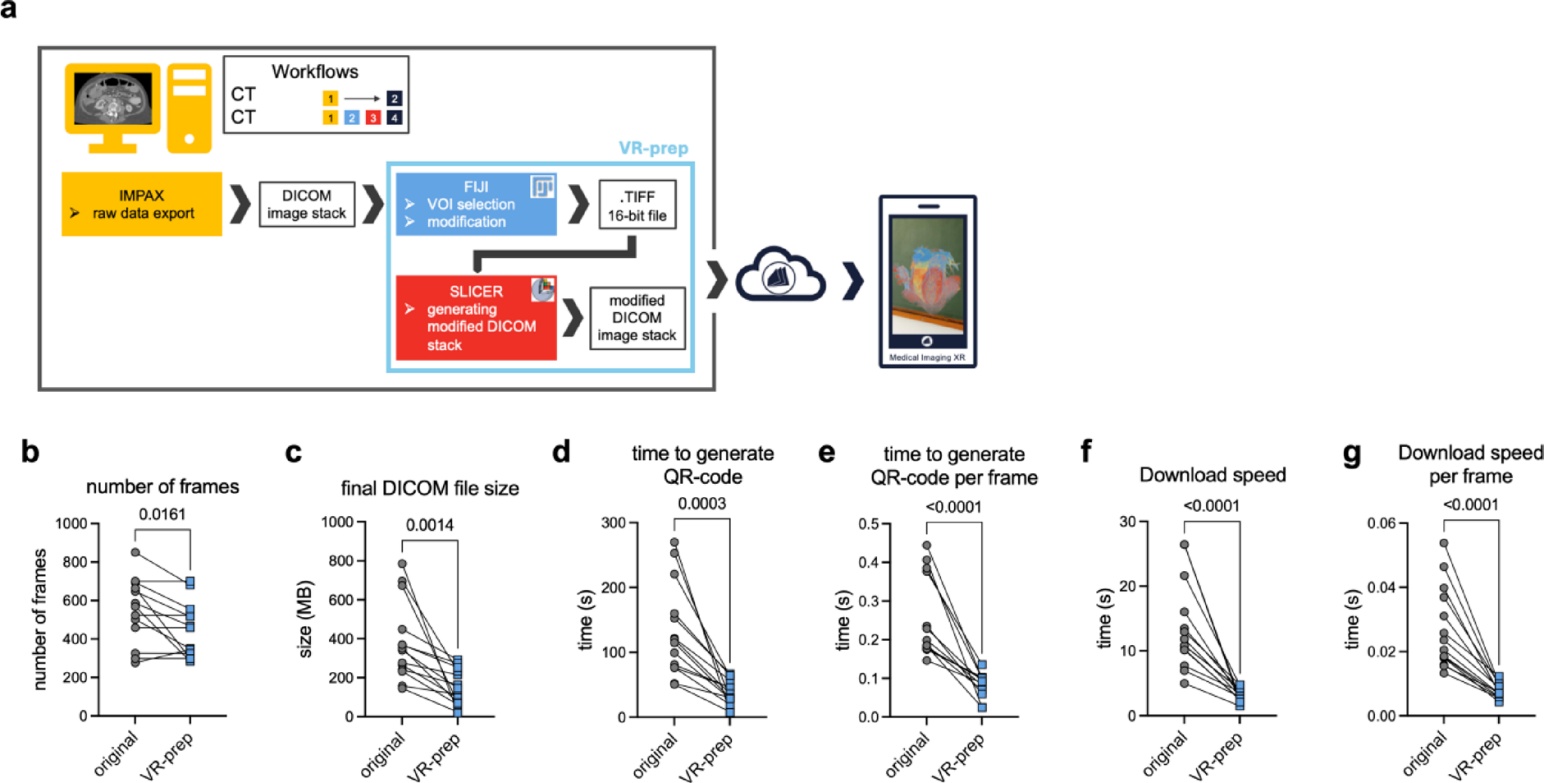
VR-prep: a workflow to accelerate and standardize the generation of 3D data sets with Medical Imaging XR. **(a)** Schematic representation of the workflow. **b)** Number of frames in the DICOM data set before and after VR-prep. **(c)** Size of the DICOM data set before and after VR-prep. **(d)** Time to generate a QR-code in Medical Imaging XR before and after VR-prep. **(e)** Time to generate a QR-code in Medical Imaging XR before and after VR-prep, normalized to frame number. **(f)** Time to download a 3D object to an iPad Pro before and after VR-prep. **(g)** Time to download a 3D object on an iPad Pro before and after VR-prep, normalized to frame number. *N* = 14, analyzed with a paired t-test.

Medical Imaging XR (MIXR) is an open-source application by Medicalholodeck™ designed for straightforward transfer of DICOM series of three-dimensional CT images into AR. Within the MIXR framework, a DICOM series is first anonymized, then uploaded to the webpage^11^, where an AR data set is generated, which in turn can be downloaded through a QR-code onto the mobile device. Direct upload of DICOM data sets, however feasible, is relatively time consuming, especially for larger data sets. Primarily to improve the speed of generation of 3D medical objects, we devised VR-prep, a workflow for image transformation using open-source products such as Fiji and 3D Slicer (Fig. 1a). VR-prep is a set of macros to be executed within Fiji (Supplementary Material 1), the newly generated data are then saved as a modified DICOM stack utilizing 3D Slicer. In brief, VR-prep in its essential form executes a reduction of file size and conversion to isotropic voxel size, and an adjustment of the slope and bit-depth of the images. The specific procedural steps of the code are explained in the methods section in detail.

Running VR-prep on a DICOM series led to a reduction of average DICOM frame number and of size of the DICOM file (435.9 ± 143.4 vs. 528.1 ± 178.3 frames (original), *p* = 0.0161 *n* = 14; 145.3 ± 94.1 MB vs. 382.2 ± 201.0 MB (original), *p* = 0.0014, *n* = 14, respectively) (Fig. 1b-c). VR-prep also led to a reduction of the time needed to upload the DICOM series to MIXR and generate a QR-code (38.1 ± 19.8 s vs. 136.1 ± 71.3 s, *p* = 0.0003, *n* = 14) (Fig. 1d). The decrease in duration was not exclusively caused by the reduction of frame number, as the normalized upload time to frame number was also reduced after VR-prep from 0.25 ± 0.10 s/frame to 0.08 ± 0.03 s/frame (*p* < 0.0001) (Fig. 1e). The download speed of the generated 3D object is also improved with use of VR-prep. ‘VR-prepped’ DICOM series were downloaded in 3.22 s ± 0.97, compared to original DICOM series 13.60 s ± 6.77 (*p* < 0.0001, *n* = 14) (Fig. 1f). Time needed for download normalized to frame number highlighted that VR-prep also allows for faster download of each individual frame (0.007 s vs. 0.027 s, *p* < 0.0001) (Fig. 1g). Overall, VR-prep allows for a faster generation of an AR object from a given DICOM series in MIXR.

## Image quality after VR-prep

**Fig. 2:**
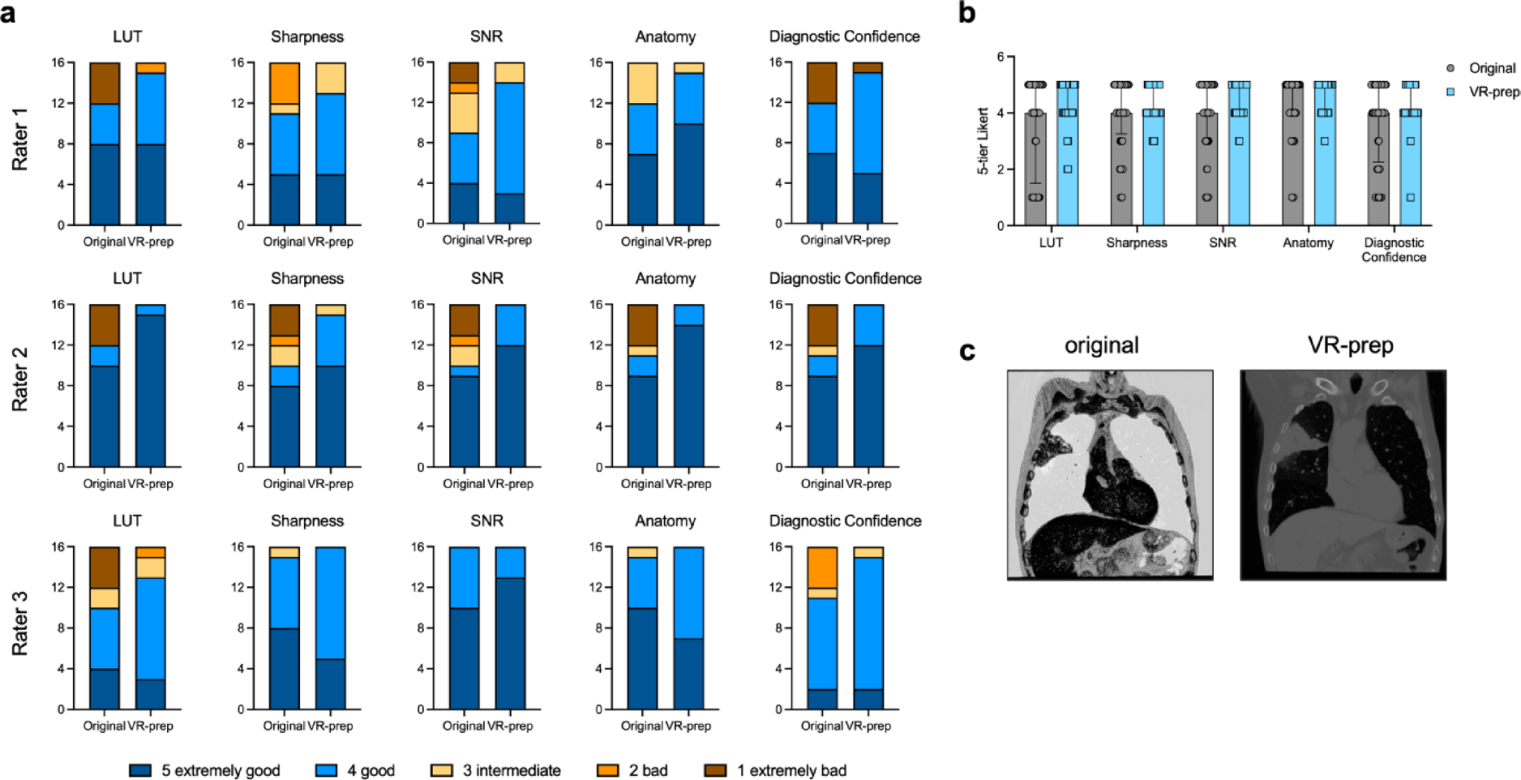
VR-prep does not impact negatively the quality of the 3D image generated in Medical Imaging XR. **(a)** Individual ratings of images uploaded to MIXR generated from the original DICOM or VR-prepped DICOM series. Each rater evaluated 32 images for their LUT, sharpness, SNR, anatomical structures (Anatomy) and confidence for use in diagnostics (Diagnostic confidence) **(b)** Combined ratings for quality of 3D objects before and after VR-prep. Data plotted corresponds to median and IQR. **(c)** Example image of a 3D stack with incorrect intensity values.

We continued with assessing whether the data transformation of VR-prep affects the overall quality of the AR images visualized in MIXR. Three independent raters evaluated VR-prepped and original images and rated using a 5-tier Likert scale for five parameters: LUT, sharpness, signal to noise ratio (SNR), anatomical structures and confidence in use for diagnostics (Fig. 2a). We evaluated intraclass correlation coefficient for all the ratings and found a higher variability in the ratings in the VR-prep images than in the original (Table 1). Combined scores from all raters were also calculated (Fig. 2b; Table 2). VR-prep improved image quality in regard to LUT representation (4 [2.5–5.5] vs. 5 [4–5], *p* = 0.0022). VR-prep also improved SNR and identification of anatomical structures by rating clinicians. Overall, these contributed to a better rating in confidence in use of VR-prepped images for diagnostics (4 [2.75–5.75] vs. 4 [4–5], *p* = 0.0073). The highest contributing factor for the inferior performance of the original images was the fact that, in 43% (6/14) of our tested cases, the generated 3D data had inverted intensity values, as exemplified in Fig. 2c. This inversion contributed to the difficulty in identifying anatomical structures, worsened sharpness and SNR and led, overall, to a lower confidence in using these AR images for diagnostics purposes.

**Table 1:** Intraclass correlation coefficient between ratings of image quality in images imported to MIXR.

	Parameters	ICC	95% CI	*p*-value
Original	Intensity	0.957	0.897–0.984	< 0.001
	Sharpness	0.64	0.216–0.860	0.005
	SNR	0.676	0.278–0.874	< 0.001
	Anatomy	0.789	0.513–0.920.513.920	< 0.001
	Diagnostic confidence	0.953	0.893–0.982	< 0.001
VR-prep	Intensity	0.506	0.002–0.798	0.006
	Sharpness	0.711	0.36–0.888	< 0.001
	SNR	0.44	−0.057–0.762.057.762	0.011
	Anatomy	0.565	0.086–0.827	0.012
	Diagnostic confidence	0.259	−0.375–0.683.375.683	0.183

ICC, intraclass correlation coefficient; CI, confidence interval.

**Table 2:** Combined evaluations of all three raters on VR-prep and original image XR images imported to MIXR.

**Parameters**	Original		VR-prep		p-value
	Median	IQR	Median	IQR	
Intensity	4	2.5–5.5	5	4–5	0.0022
Sharpness	4	3.75–5.75	4	4–5	0.1715
SNR	4	3–5	5	4–5	0.0036
Anatomy	5	4–5	5	4–5	0.0183
Diagnostic confidence	4	2.75–5.75	4	4–5	0.0073

Statistics were evaluated with Wilcoxon test. IQR, interquartile range. SNR, Signal to Noise ratio.

## VR-prep vs. increased step size of the original DICOM series

**Fig. 3:**
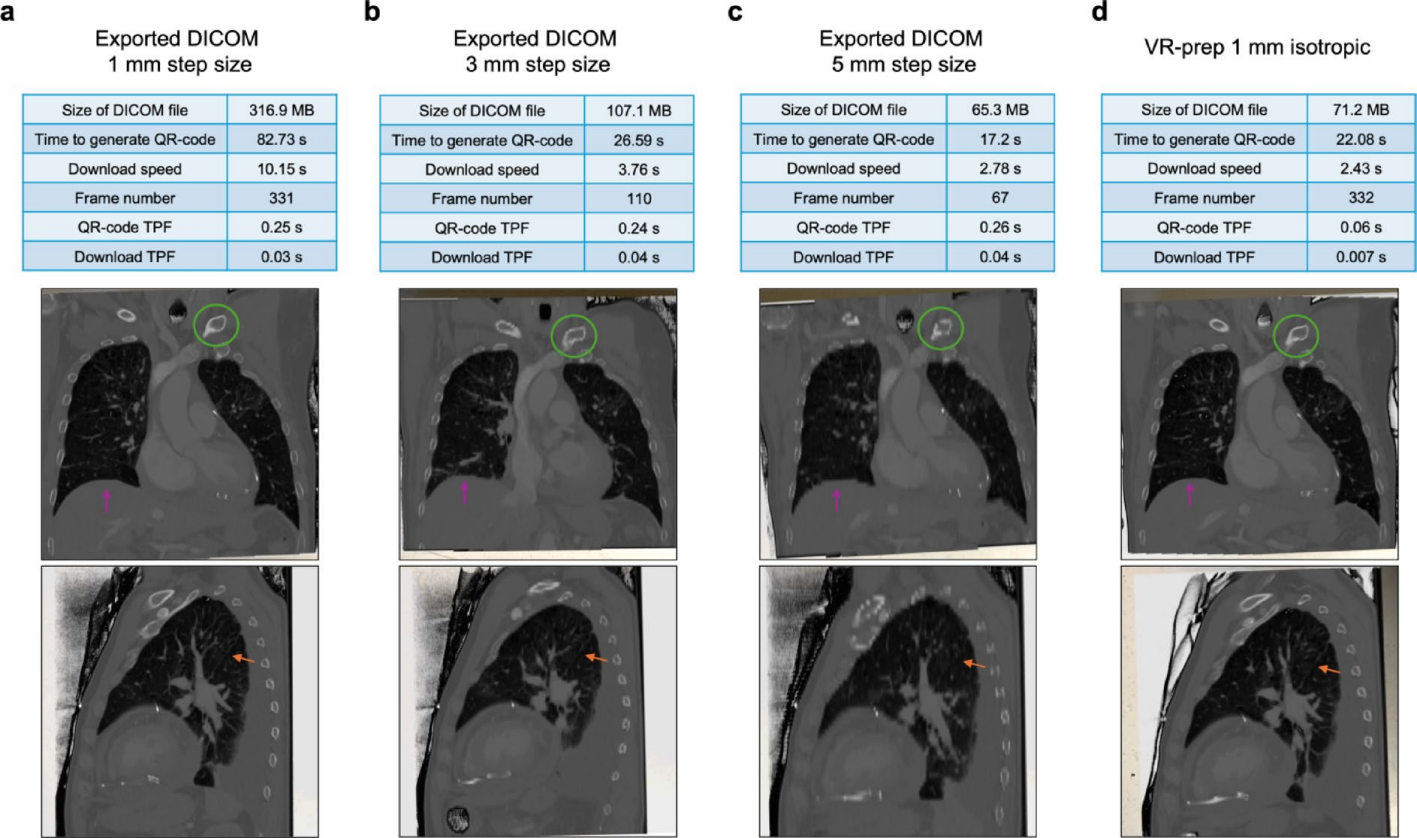
VR-prep results in superior 3D objects than exporting DICOM series at lower resolution. The same image series was exported at 1, 3, and 5 mm increment from PACS and the 1 mm increment was used to run VR-prep. Image specifications for DICOM series exported at **(a)** 1 mm, **(b)** 3 mm, **(c)** 5 mm, and **(d)** VR-prep. Green circle highlights the left clavicle; purple arrows points at diaphragm; orange arrow points at the interlobar fissure of the left lung. TPF: time per frame.

Next, we aimed to investigate whether original image stacks reconstructed in the scanner with a larger increment (e.g. a 5 mm increment compared to our diagnostic standard of 1 mm) would be sufficient to overcome the acceleration provided by VR-prep utilizing a 1 mm increment ground data. To test this, we proceeded by comparing VR-prep with DICOM series containing larger increment (Fig. 3). The size of the DICOM file, the time to generate a QR-code and the download speed were reduced with VR-prep to a similar magnitude of the 5 mm DICOM series, maintaining a high frame number, equivalent to the original DICOM series. Time for QR-code generation per frame was maintained in all DICOM data sets 0.25 s, 0.24 s, and 0.26 s, except for the VR-prep image, with a much lower value of 0.06 s per frame. The same effect was seen in the downloading speed to an iPad Pro, with VR-prep recording a much faster download speed of 0.007 s per frame, compared to 0.3 s, 0.4 s, 0.4 s of the DICOM series exported from PACS. Another relevant factor is considered to be the image quality of 3D object: VR-prepped DICOM series provides a comparable image quality to the original 1 mm DICOM series with sharp contrast and clearly defined contouring of anatomical structures, such as the left clavicle, the diaphragm and the interlobar fissure of the left lung (Fig. 3). The 3D images created from image original series at 3 and 5 mm increment yielded insufficient sharpness and overall image clarity, rendering them unsuitable for clinical evaluation. Overall, VR-prep improves AR practicability, as it speeds up the generation of 3D objects, without compromising image quality and readability.

## Integration of diverse medical image series

**Fig. 4:**
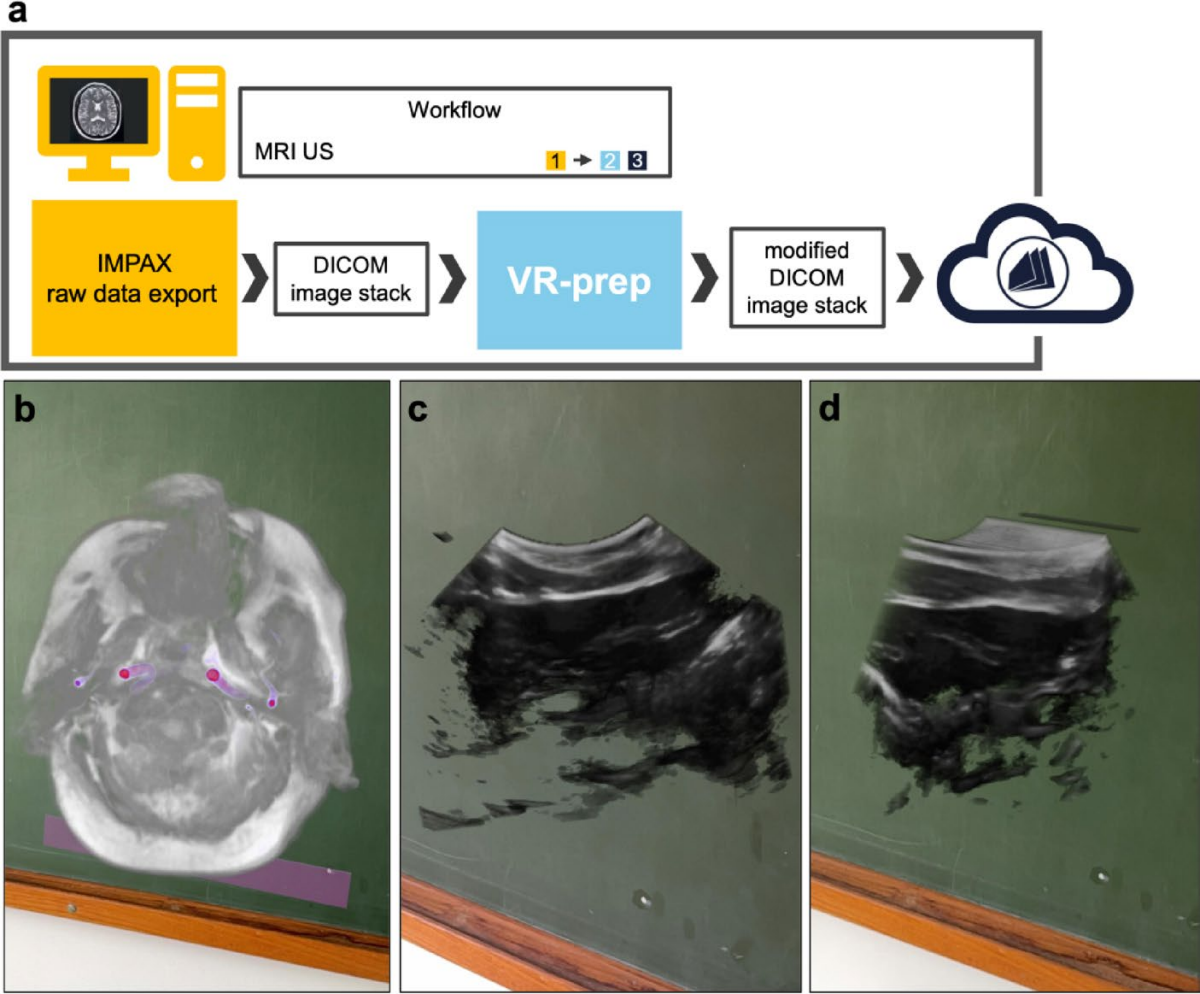
Workflow for the visualization of MRI and ultrasound data with VR-prep and Medical Imaging XR. **(a)** Demonstration of incorporation of VR-prep in the workflow. IMPAX as synonym for Picture Archiving System. **(b)** AR image displays an MRI angiography of the head/neck region (grayscale) enhanced by contrast media (purple). **(c-d)** The AR images display an ultrasound image stack at two different orientations. Note that the AR image on the right side is cropped in real-time via Medical Imaging XR.

We continued by testing VR-prep in different data sets than CT DICOM series. First, we used VR-prep to prepare MRI data series and visualize it in MIXR (Fig. 4a-b). We have also tested non-DICOM medical image series, which is the case of ultrasound (US). We successfully converted the time dimension into a pseudo-volume dimension and run VR-prep. The result image imported into MIXR is shown in Fig. 4c-d.

**Fig. 5:**
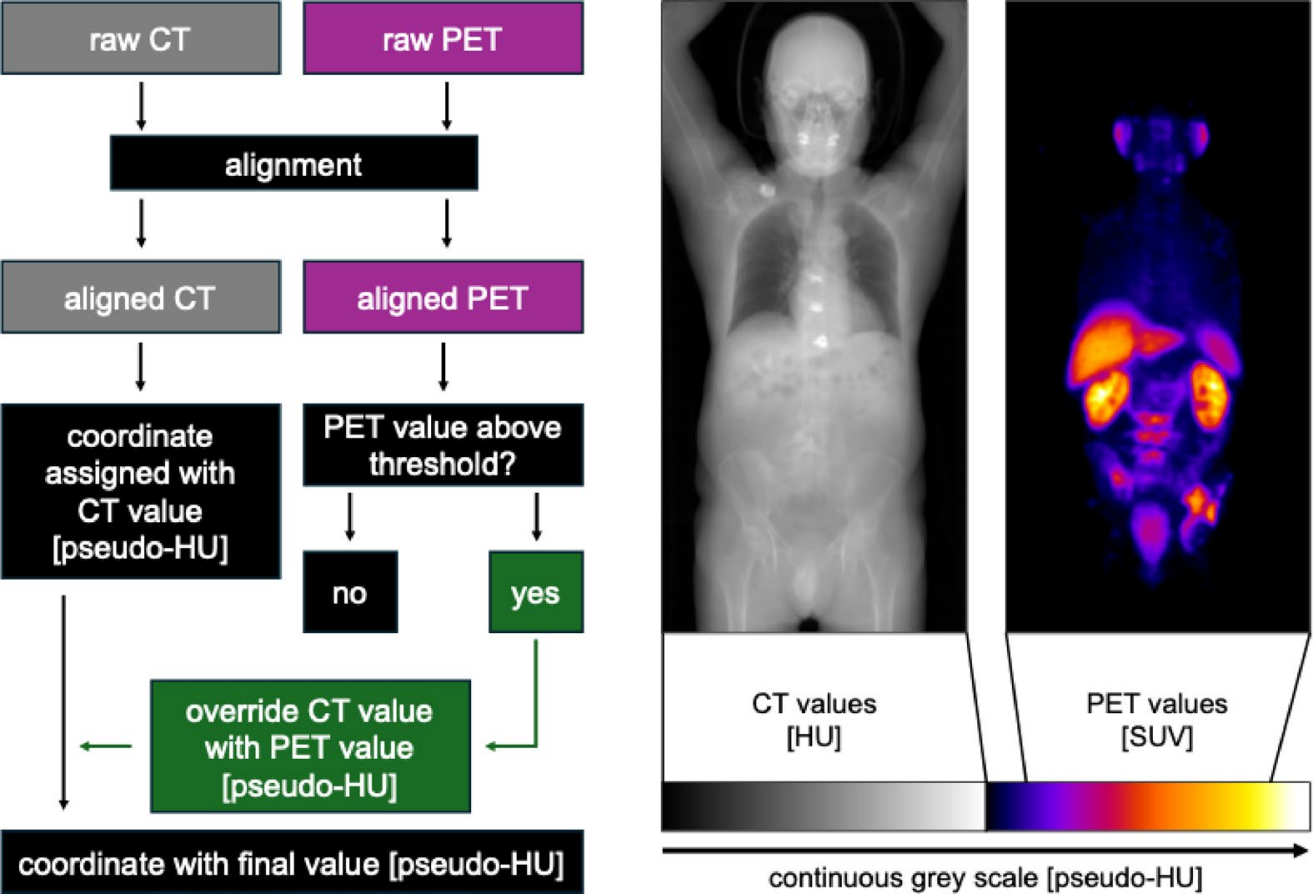
Raw CT and PET images were aligned to ensure voxel-wise correspondence. For each coordinate, two possible assignments were defined: if the PET signal was below threshold, the coordinate retained its CT-derived Hounsfield Unit (HU); else the PET signal exceeded threshold, the CT value was overridden and replaced with the PET-derived value (alternative path shown in green). This process yielded a final integrated dataset in pseudo-HU, combining both structural and functional information. Representative images illustrate aligned CT (left) and PET (right) values with corresponding scales.

**Fig. 6:**
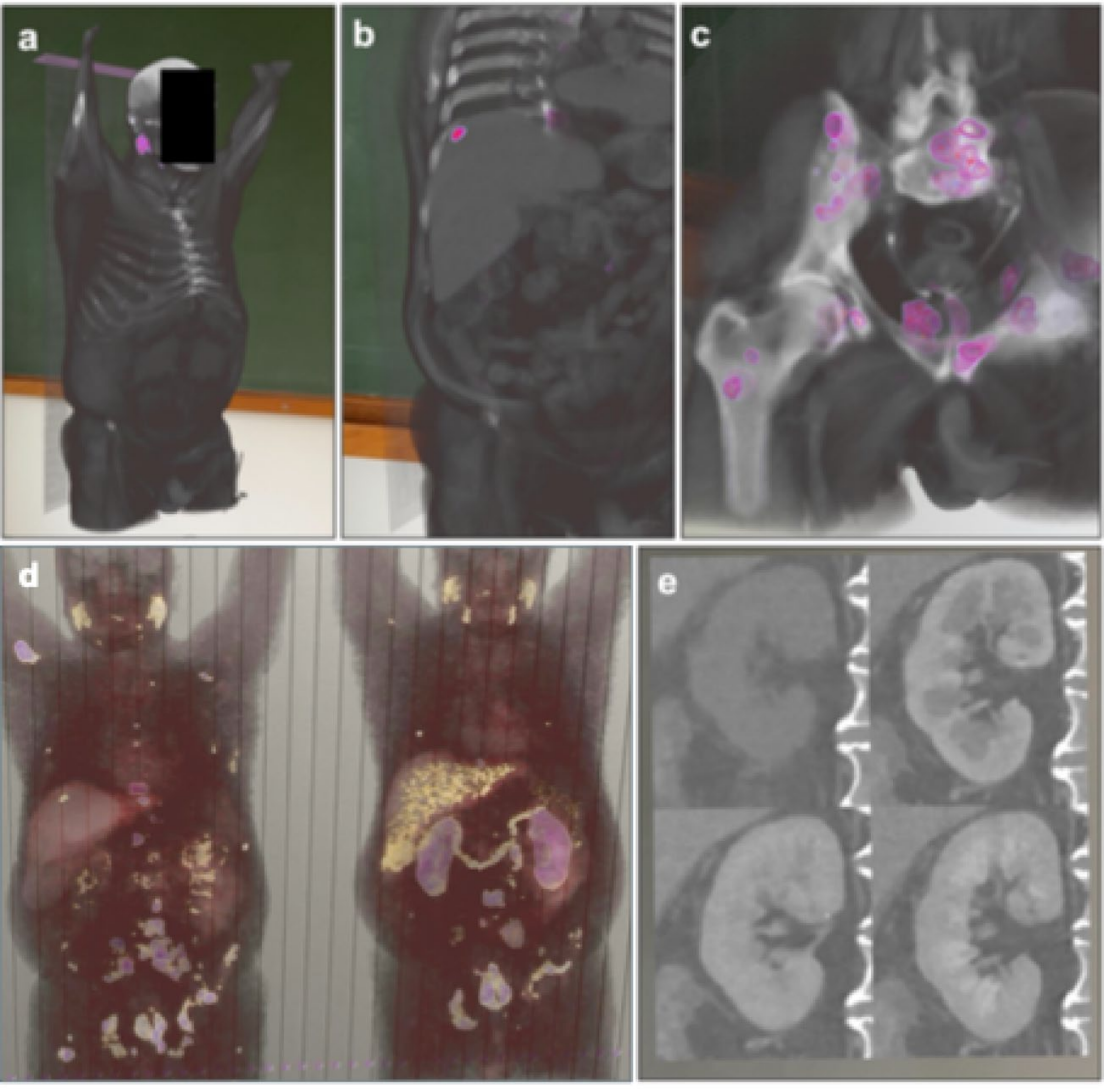
Integration of PET-CT and different time points using VR-prep. **(a)** AR images depict the entire scan volume with anonymized facial characteristics, **(b-c)** real-time cropping of the AR volume exposing a liver metastasis **(b)** and the pelvic tumor load **(c)**. **(d-e)** Combination of scans from different time points in the same AR object. **(d)** PSMA PET patient before (left) and after (right) treatment show reduction of metastases. **(e)** right kidney of one patient at different time points: no contrast media (top left), arterial (top right), portal venous (bottom left) and venous (bottom right) phase of the contrast media passage.

To visualize CT and PET signal in one stack we designed another macro for Fiji (Supplementary Material 2), which builds up on our first macro which integrates the intensity values of the CT and PET signals into a single.tiff file, as described in detail in the methods section and Fig. 5. The results were transformed into DICOM format using 3D Slicer and imported into MIXR (Fig. 6a-c, Sup. Video 1). Next, we tested displaying comparative 3D samples into AR. First, we chose to compare PET data sets for a patient with metastasized prostate cancer before and after treatment (Fig. 6d, Sup. Video 2). Second, we used images of a kidney in a native as well as three different contrast phases (Fig. 6e, Sup. Video 3).

**Fig. 7:**
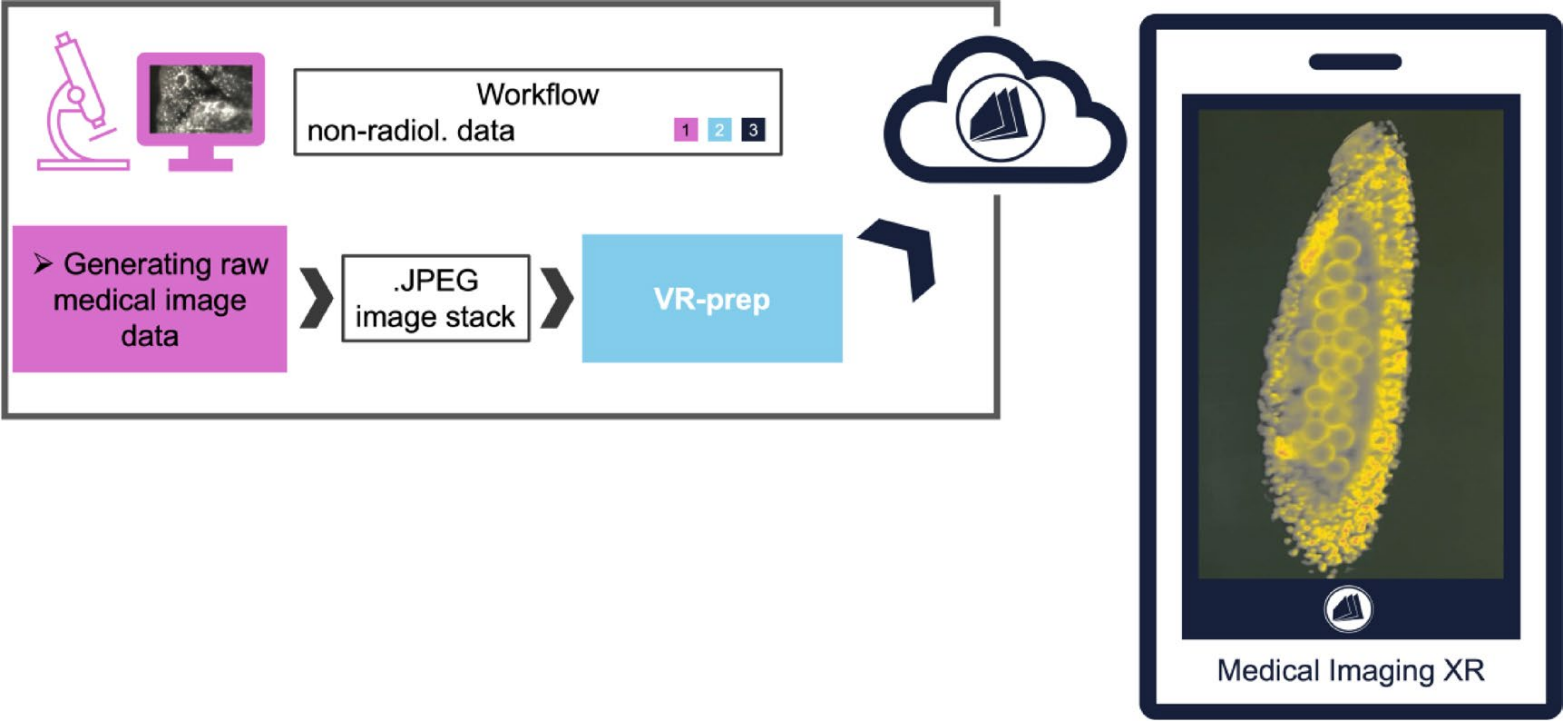
Workflow for the visualization of microscopy data with VR-prep and Medical Imaging XR. The AR images display a portion of a flower bud.

Lastly, we aimed to extend the usability of our framework to other medical image data, particularly non-radiological. We chose to use Light-sheet-fluorescence-microscopy (LSFM) imaging and procured a publicly available 3D data set of a flower bud lobe^12^. LSFM allows for high resolution imaging while maintain a depth dimension. The downloaded images in.png format were straightforwardly imported to Fiji and processed further with VR-prep (Fig. 7, Sup. Video 4). In result, VR-prep allows for integration of different clinically relevant image modalities in MIXR, contributing to the larger application of AR in clinical practice and decision making. We also open the door for non-clinical scientific data to be explored using MIXR, broadening the range of applications for smartphone/tablet-based AR.

## Discussion

AR technology holds promise in revolutionizing the way medical professionals and patients interact with image data^1,13^. Particularly in the collaboration between radiology and other disciplines, AR offers valuable tools for visualizing complex anatomical structures in three dimensions, facilitating better understanding and planning of medical procedures^7,14^. However, widespread implementation of AR frameworks in clinical practice has been hampered by administrative hurdles related to data protection, high equipment and infrastructure costs, and the need for specialized training. To exemplify this with regards to the equipment, many research facilities rely on expensive head-mounted-displays^4,5^, which themselves require a substantial financial and time effort to achieve an operational status. As smartphones and other mobile devices are widely spread and possess increasingly strong computational capabilities the usage of smartphones for AR is appealing.

To assess the principal usability of AR in clinical routine, we first explored app-based solutions for mobile devices. Previously, the use of smartphones and tablets for AR guided clinical procedures, for example for percutaneous nephrolithotomy, have been explored^15,16^. Furthermore, workflows are being devised for surgery planning^17^. In the field of medical education, there is increasing interest in using smartphone based AR approaches^18,19^. We intend to implement AR in our clinical routine with Medical Imaging XR (MIXR), an open-source software provided by Medicalholodeck™. MIXR, like other tools, holds promise for the integration of medical imaging in portable devices but, to our knowledge, has not yet been tested for case demonstration between clinicians or medical education. This might be due to the limited range of data that can be displayed, in particular, data size constraints and a limited variety of image modalities. We conceptualized VR-prep to increase the range of medical imaging modalities displayable within MIXR but can likely be deployed to other similar AR platforms. The macros constituting VR-prep run exclusively in the open-source software suit Fiji, making it easy and unexpensive to deploy. Afterwards the generated image stacks can be transformed into a DICOM image series with the also open-source 3D Slicer.

In general, our VR-prep workflow reduced the complexity of the DICOM series significantly, without impacting AR image quality, as determined by medical professionals. Because smartphones and tablets are, relatively to head mounted displays for example, limited in computational power, the file size reduction boosts the rendering performance and aids the overall stability of the AR app. In principle, implementing VR-prep for DICOM series at the time of export from the scanner (or alternatively as an integrated tool within the radiological image assessment platforms) would allow for direct AR visualization in a clinical setting. This holds potential for disciplines relying on radiological data for pre-interventional planning. A widespread and timely distribution of spatial data may also positively impact planning efficiency and efficacy. However, it must be clearly stated that neither MIXR, nor our contribution with VR-prep, is licensed as a medical product. Hence, the deployment of this technology currently acts as an add-on to the gold standard of clinical routine.

Another product of our study is a separate macro for Fiji, which allows for swift data alignment of PET-CT scans, paving the way for integrating the relevant data from both modalities into a single AR compatible image stack, which we described in detail to foster the implementation by other groups. Despite demonstrating the feasibility of the approach for PET-CT, we acknowledge that further steps towards automatization are required.

In practicality, from the time one has exported an image series from the radiological software and the subsequent image modifications and VR-prep are done, it takes less than 1 min to visualize the data set in AR. The relative lower time achieved by VR-prep facilitates usage in clinical practice, where faster protocols are preferred by clinicians. Importantly, the image modifications that allow for a faster image import and export do not compromise the quality of the AR image itself. We also emphasize that any image data can be channeled into our workflow, if it can be imported into Fiji. To this end, specific Fiji plugins are available to extend compatibility to over 130 file formats^20^.

Our work has limitations though: We tested VR-prep in a relatively low number of CT scans. Nevertheless, we tested a wide variety of image modalities including CT, MRI, US, PET-CT and 3D-Microscopy as well as different organs and organ systems and range of voxel sizes within the foundational data sets. Another limitation from our work is the fact that we focused our performance assessment on CT data and hence did not obtain the same metrics with MRI or US series. We did not overserve any problem with images from any image modality using VR-prep, as the final modified DICOM series in 3D Slicer is generated to be a CT series, regardless of the original data set.

Another limitation is the low number of evaluators for image quality metrics. The clinicians that evaluated the images showed a wide variety of responses in regard to image quality. We can only hypothesize that this could be due to different levels of previous contact with AR tools, and different levels of expectations regarding image clarity. A higher number of evaluators could minimize these effects.

Although we achieved good quality, 3D renderings of several types of medical imaging data sets like CT, MRI and PET-CT, ultrasound remains challenging. Ultrasound videos must fulfill certain quality criteria to generate informative 3D AR objects. To this end, the operator must move the probe unidirectionally and uniformly while maintaining the angle. Imperfections in recording will directly translate to a distorted and potentially misleading AR visualization. This ultimately means that most US videos that have been recorded for diagnostic purposes are not compatible with representation in AR.

In conclusion, VR-prep represents an effort in democratizing the field of medical AR, offering a cost-free and user-friendly solution for visualizing complex medical imaging data in research and illustrates the potential for clinical practice, although several hurdles must still be overcome towards clinical implementation. Taken together, by addressing the administrative, financial, and training barriers associated with traditional AR implementations through technical innovation, VR-prep may prove helpful to transform the way healthcare professionals interact with patient data and ultimately improve patient care and outcomes.

## Materials and methods

### Patients

Patient DICOM series were procured from our local Picture Archiving System (PACS). The study was approved by the institutional review board (IRB University Hospital Bonn, 303/16). Due to the retrospective nature of the study, the IRB waived the need of obtaining informed consent. The study was performed in accordance with the relevant guidelines and regulations including the Declaration of Helsinki.

## Raw image data (CT cases used for benchmarking)

The CT imaging datasets used in this study consisted of volumetric scans acquired with variable technical parameters. The number of slices per volume ranged from 205 to 850, reflecting differences in anatomical coverage and acquisition settings. Field of view dimensions spanned from smaller regions of 176–186 mm to extended coverage exceeding 490 mm, while the in-plane matrix size varied between 512 × 512, 768 × 768, and, in one instance, a higher resolution acquisition of over 2000 × 1900 pixels. Voxel resolutions demonstrated a wide spectrum, with the smallest sampling at 0.1 × 0.1 mm in-plane and slice thickness below 1 mm, and the largest spanning over 1 mm in-plane with slice thickness up to 1.5 mm. A description of the cases regarding scan regions and pathological findings is included in Table 3.

**Table 3:** Description of cases used to test VR-prep.

Scan area	*N* cases	Pathological findings
Thorax	5	Pneumonia, emphysema, aortic stenosis, and cancer
Abdomen	5	Pancreatitis, intestinal herniation, ovarian teratoma, and splenic aneurism
Extremities	3	Fracture, sarcoma, and arthrosis
Full body	3	Cancer and polytrauma

### Imaging devices

Experiments have been carried out with data from a photon counting CT (NAEOTOM Alpha, Siemens Healthineers, Germany), a 3 T MRI (CIMA.X, Siemens Healthineers, Germany), an ultrasound (Epiq 5G, Philips Healthcare, the Netherlands), and a PET/CT (Philips Vereos, Philips Healthcare, the Netherlands).

### Computational setup

The radiological desktop computer was a Hewlett-Peckard HP Z620 Workstation with an Intel Xeon CPU E5-2620 with 6 cores and 2.0 GHz, RAM of 16 GB, solely tasked with raw data anonymization and export. The workstation ran on a Microsoft Windows 10 Education operating system. Processing of priorly anonymized raw data was executed exclusively using an Apple MacBook Air with a M1 processor, 8 GB RAM and macOS 14.4.1. No GPU acceleration was used, despite the Apple M1 processor, because Fiji does not utilize GPUs in this build version. The task performed in 3D Slicer was also carried out without GPU acceleration.

### Mobile device

For all displays of AR data an Apple iPhone 13 mini with 128 GB storage running on iOS version 17.4.1 and an iPad Pro with 1 TB storage running on iPadOS version 17.5.1 were used. We successfully tested the principal software compatibility on iPhone 7, iPhone 14 and iPhone 15 Pro.

### Fiji (Fiji is just ImageJ)

Fiji Version 2.14.0/1.54f, an open-source platform built on ImageJ, was used for processing image data^10^. All mathematical operations were carried out within Fiji.

### 3D Slicer

3D Slicer (Version 5.0.3) is an open-source software platform for medical image informatics, image processing, and three-dimensional visualization. Herein, the ‘Create a DICOM Series’ was exploited to generate new DICOM series.

### Medical Imaging XR

Medical Imaging XR (Version 2.6.8) is an open-source program, provided by ‘nooon WEB&IT GmbH’ and under the copyright of Medicalholodeck™. The software is capable to display DICOM stacks after cloud processing in AR. The user can adjust the LUT freely and crop the sample in real-time in relation of the mobile device to the projected sample.

### VR-prep for single modality image stack generation

VR-prep uses Fiji to convert a DICOM data set (or other formats) into a.tiff file. The intensity values are verified, converted to a positive range, converted to isotropic 1 mm resolution, and finally, the series is converted to an unsigned 16-bit. The image series must initiate caudally and move cranially. Subsequent image stacks are a single .tiff file encompassing the entirety of the individual images. This.tiff file is imported to 3D Slicer to generate a DICOM series with the plugin “Create DICOM series”, that can be uploaded to Medical Imaging XR, for 3D object generation. Time need to upload the DICOM series to generate the QR code and to upload the AR model to the app on the tablet was measured with a stop watch.

### VR-prep for combining different modality image stacks into a single VR-object

CT and PET images were first acquired independently and underwent the VR-prep operations within Fiji. The resulting two .tiff image stacks were subsequently aligned to ensure spatial correspondence between the two modalities. Following alignment, each voxel was initially assigned a CT-derived pseudo-Hounsfiled-Unit [pseudo-HU]. In particular, CT values were compressed into a 0–499 value range, scaling linearly in the range from − 1000 to + 1000 HU. Values below or in excess of this interval were assigned the pseudo-HU value 0 (for values <−1000 HU) or 499 (for values > + 1000 HU). PET values were then evaluated against a predefined threshold, and for voxels exceeding this threshold, a pseudo-HU was determined scaling linear to the individual set threshold (500 pseudo-HU) to a standard-uptake-value of 100 Bq/mL (defined as 999 pseudo-HU). For each coordinate, the CT-derived pseudo-HU value was initially set. If for a given coordinate the PET-derived signal exceeded the priorly mentioned threshold, the CT-derived value was overridden with the PET-derived value, mapped into the pseudo-HU space. This process produced a final integrated image stack in which each voxel contained a continuous pseudo-HU value, enabling visualization and quantitative analysis of both structural and functional information within a single dataset. Figure 6 illustrates the operations of this procedure schematically.

### Quantification of performance

To evaluate the performance of VR-prep, we uploaded the original DICOM series as well as the VR-prep DICOM series to Medical Imaging XR and quantified the time. Following, we quantified the time to download the 3D object into an iPad Pro running on iPadOS version 17.5.1. For quality assessment of AR images, 3 radiologist with 4 (AMCB), 1 (MB) and 1 (LO) years of experience evaluated five parameters: adequacy of look up tables generated (labeled as LUT), sharpness, signal to noise ratio (SNR), possibility of correct identification of anatomical structures (labeled as Anatomy), and confidence in use of the generated AR image in diagnostic configurations (labeled as Diagnostic confidence). A 5-tier Likert scale was used with the following descriptors: 1 - extremely bad, 2 - bad, 3 -intermediate, 4 - good, and 5 - extremely good. All evaluators analyzed all images, blinded and at a random order. They were allowed as much time per image as they deemed necessary and were allowed to use all functions of the app, including change of intensity values, crop, and zoom.

To compare VR-prep performance with original DICOM series exported with different frame increments, we reformatted in PACS an image series to different slice thicknesses (3 and 5 mm) and used the original 1 mm to run VR-prep. The original image series was acquired at 1 mm increment, with a voxel size of 0.5566 × 0.5566 × 0.9990 mm^3^, the VR-prepped series had a voxel size of 1 × 1 × 1 mm^3^, and the reformatted DICOM series to 3 and 5 mm had a voxel size of 0.5594 × 0.5594 × 3.0057 mm^3^ and 0.5594 × 0.5594 × 4.9639 mm^3^, respectively. Duration of upload of DICOM series to MIXR and duration of download to the mobile device were quantified.

### Integration of MRI and microscopy in VR-prep

An MRI DICOM series was procured on our PACS data base. The MRI DICOM series can be directly run in VR-prep.

For microscopy data set we downloaded a publicly available data set of a flower bud lobe, acquired with a light-sheet-fluorescence-microscope^12^. The downloaded images in .png format were imported to Fiji, converted into .tiff and processed further with VR-prep.

### Integration of ultrasound in VR-prep

Ultrasound data sets are not DICOM series, and are typically a colored .jpeg. Images are acquired continuously with a probe, hence the time dimension is added at the expense of the stack-depth dimension. By following a stringent path at a stable speed using the probe, the operator can capture an anatomical region in pseudo-3D. We opened the .jpeg data set in Fiji, converted into.tiff and processed further with VR-prep.

### Integration of PET-CT in VR-prep

PET and CT data set must be integrated in the same DICOM series to be transferred to MIXR. With this in mind, we adjusted in Fiji the intensity values of the CT series to a range of 0 to 100 and the PET series to a range of 105 to 255, in 8-bit. Series were added to each other and run in VR-prep.

### Integration of time comparison 3D images

To combine image series of different time points side by side, we used Fiji command “combine stack”. Then, images were run in VR-prep.

### Statistical analysis

GraphPad Prism (Version 10.4.0), developed by GraphPad Software, LLC, was used for the statistical evaluation. Significance was set to a p-value lower than 0.05 for a paired t-test. Results of the Likert scale are presented as median and Interquartile Range (IQR). The evaluation of the 5-tier Likert scale was calculated with GraphPad Prism (Version 10.4.0). Median and IQR were analyzed with Wilcoxon test and statistical significance was set to 0.05. ICC was calculated using SPSS, with a k = 3 and 95% CI.

### Manuscript writing

We acknowledge the use of ChatGTP 3.5 for proofreading our manuscript.

## Supplementary Information

Below is the link to the electronic supplementary material.

Supplementary Material 1

Supplementary Material 2

Supplementary Material 3

Supplementary Material 4

Supplementary Material 5

Supplementary Material 6

## Data Availability

Raw data and final DICOM series that were uploaded to Medical Imaging XR will be provided upon reasonable request to the corresponding author AMCB.

## References

[1] Tortora, M. et al. Current applications and future perspectives of extended reality in radiology. *Radiol. med.* **130**, 905–920 (2025). 40153208 10.1007/s11547-025-02001-2 PMC12185561

[2] Jang, Y. et al. Clinical application of an augmented reality navigation system for transforaminal epidural injection: A randomized controlled trial. *J. Clin. Med.* **13**, 1992 (2024). 38610758 10.3390/jcm13071992 PMC11012780

[3] Wehrkamp, K. et al. The impact of Virtual-, Augmented- and mixed reality during preoperative informed consent: A systematic review of the literature. *J. Med. Syst.* **49**, 89 (2025). 40555846 10.1007/s10916-025-02217-9 PMC12187789

[4] Lauinger, A. R. et al. Applications of mixed reality with medical imaging for training and clinical practice. *JMI* **11**, 062608 (2024). 39734608 10.1117/1.JMI.11.6.062608 PMC11669596

[5] Iqbal, A. I. et al. Immersive technologies in healthcare: an In-Depth exploration of virtual reality and augmented reality in enhancing patient Care, medical Education, and training paradigms. *J. Prim. Care Community Health*. **15**, 21501319241293311 (2024). 39439304 10.1177/21501319241293311 PMC11528804

[6] Looser, J. *AR Magic Lenses: Addressing the Challenge of Focus and Context in Augmented Reality* (University of Canterbury, 2007).

[7] Arensmeyer, J. et al. A System for Mixed-Reality Holographic Overlays of Real-Time Rendered 3D-Reconstructed Imaging Using a Video Pass-through Head-Mounted Display—A Pathway to Future Navigation in Chest Wall Surgery. *JCM* **13**, (2024). (2080). 10.3390/jcm13072080 PMC11012529 38610849

[8] Feodorovici, P. et al. Collaborative virtual reality Real-Time 3D image editing for chest wall resections and reconstruction planning. *Innovations (Phila)*. **18**, 525–530 (2023). 38073259 10.1177/15569845231217072

[9] Jacob, A. M. et al. Augmented reality live demonstrations during traditional lectures improve Understanding of computed tomography data sets by medical students. *Front Virtual Real* **6**, (2025).

[10] Schindelin, J. et al. Fiji: an open-source platform for biological-image analysis. *Nat. Methods*. **9**, 676–682 (2012). 22743772 10.1038/nmeth.2019 PMC3855844

[11] Medicalholodeck Medical Imaging XR - Upload Page for DICOM Files.

[12] Valuchova, S. et al. Imaging plant germline differentiation within Arabidopsis flowers by light sheet microscopy. *eLife* **9**, e52546 (2020). 32041682 10.7554/eLife.52546 PMC7012603

[13] Kanschik, D., Bruno, R. R., Wolff, G., Kelm, M. & Jung, C. Virtual and augmented reality in intensive care medicine: a systematic review. *Ann. Intensive Care*. **13**, 81 (2023). 37695464 10.1186/s13613-023-01176-z PMC10495307

[14] Bocanegra-Becerra, J. E. et al. Toward a frontierless collaboration in neurosurgery: A systematic review of remote augmented and virtual reality technologies. *World Neurosurg.* **187**, 114–121 (2024). 38636636 10.1016/j.wneu.2024.04.048

[15] Rassweiler-Seyfried, M. C. et al. iPad-assisted percutaneous nephrolithotomy (PCNL): a matched pair analysis compared to standard PCNL. *World J. Urol.* **38**, 447–453 (2020). 31073641 10.1007/s00345-019-02801-y

[16] Saccenti, L. et al. Integrated needle guide on smartphone for percutaneous interventions using augmented reality. *Cardiovasc. Intervent Radiol.* **48**, 1042–1052 (2025). 40295398 10.1007/s00270-025-04044-4 PMC12267309

[17] Necker, F. N. et al. Leveraging the Apple ecosystem: easy viewing and sharing of Three-dimensional perforator visualizations via iPad/iPhone-based augmented reality. *Plast. Reconstr. Surg. Glob Open.* **12**, e5940 (2024). 38957720 10.1097/GOX.0000000000005940 PMC11216661

[18] Sveinsson, B., Koonjoo, N. & Rosen, M. S. ARmedViewer, an augmented-reality-based fast 3D reslicer for medical image data on mobile devices: A feasibility study. *Comput. Methods Programs Biomed.* **200**, 105836 (2021). 33250281 10.1016/j.cmpb.2020.105836

[19] Jain, N., Youngblood, P., Hasel, M. & Srivastava, S. An augmented reality tool for learning Spatial anatomy on mobile devices. *Clin. Anat.* **30**, 736–741 (2017). 28631297 10.1002/ca.22943

[20] Fiji Plugin - OME-Bio-Formats.

